# Supplementary material for: The SOS response-associated peptidase (SRAP) domain of YedK catalyzes ring opening of abasic sites and reversal of its DNA–protein cross-link
Source: J Biol Chem. 2022 Aug 5;298(9):102307. doi: 10.1016/j.jbc.2022.102307 (PMC9436759; doi:10.1016/j.jbc.2022.102307)
Supplement: Supplemental Figures S1–S5 and Tables S1–S3 [file mmc1.pdf]

Supporting Information

The SOS response-associated peptidase (SRAP) domain of YedK catalyzes ring opening of abasic sites and reversal of its DNA-protein crosslink

Katherine A. Paulin[1], David Cortez[2], and Brandt F. Eichman[1,2,*]

[1] Department of Biological Sciences, Vanderbilt University, Nashville, TN 37232 USA

[2] Department of Biochemistry, Vanderbilt University School of Medicine, Nashville, TN 37232 USA

Supporting Methods

ESI-MS of YedK mutants. High resolution mass spectra were acquired using a Thermo XExactive quadrupole/orbitrap mass spectrometer equipped with an *Ion Max* heated electrospray ion source. The instrument was calibrated weekly over a mass range of *m/z* 195 to *m/z* 1922 using the manufacturer's recommended calibration procedure. The MS ion source was operated in positive ion mode using the following ESI parameters: sheath gas (N_2) 40; auxiliary gas (N_2) 10; capillary temp 320 °C; spray voltage 4.0 kV, auxiliary gas temp 200 °C. The mass analyzer was operated at a resolving power of 120,000 (set at 200 *m/z*). Mass spectra were acquired over a mass range of *m/z* 500 - 2000 with an AGC target of 3.0×10^6 and a maximum inject time of 200 ms. Exact masses of intact proteins were calculated from the most intense ^{13}C-isotopologues (M+16) of the most intense charge state envelopes using the following expression: $m/z = [M + 16 (1.0034) + z(1.0073)] / z$. Data collection and processing were performed using XCalibur v. 4.1 and Exactive Tune v.2.9. The mass spectrometer was interfaced with a Vanquish Horizon ultra-high performance LC system (Thermo) consisting of a binary pump, refrigerated autosampler, and a thermostated column heater. Intact proteins were chromatographically resolved at room temperature using a Waters Symmetry Shield 300™ C18 reverse phase column (3.5 µm, 2.1 × 100 mm) operating at 300 µl/min. Mobile phases consisted of 0.05% TFA (*v/v*) and 0.2% formic acid (*v*/v) in solution A (H_2O/CH_3CN, 90:10) and in solution B (2-Propanol/CH_3CN/H_2O, 50:40:10). Gradient conditions were 0–2 min, 0% B; 2–8 min, 0–100% B; 8–10 min, 100% B; 10–10.5 min, 100–0% B; 10.5–15 min, 0% B. The injection volumes were 10 µl, and the autosampler injection valve and sample injection needle were flushed and washed with a methanol/water solution between each injection.

Supporting Figures

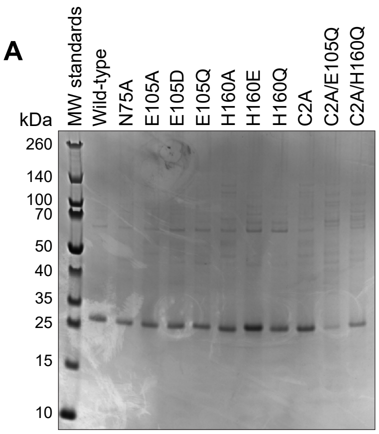

B

YedK	*m/z*	*z*	Intact $[M+H]^+$		Error (ppm)
			Deconvoluted	Theoretical	
WT	1066.7164	24	25,561.97	25,561.85	4.7
N75A	1064.9248	24	25,518.97	25,518.84	5.2
E105A	1064.2992	24	25,503.96	25,503.84	4.6
E105D	1066.1327	24	25,547.96	25,547.83	5.2
E105Q	1163.5534	22	25,560.97	25,560.87	3.8
H160A	1160.5974	22	25,495.94	25,495.83	4.1
H160E	984.4314	26	25,553.98	25,553.83	5.8
H160Q	1023.7285	25	25,552.98	25,552.85	5.2
C2A	1111.6600	23	25,529.97	25,529.88	3.3
C2A/E105Q	983.4721	26	25,529.04	25,528.89	5.8
C2A/H160Q	1022.4494	25	25,521.01	25,520.88	4.9

Figure S1. Purity and mass analysis of YedK mutant proteins. **A**. Coomassie stained SDS-PAGE gel of purified YedK proteins. **B**. ESI-MS data for purified YedK proteins. Theoretical masses were calculated without Met1. Detailed protocol is provided under *Supporting Methods*.

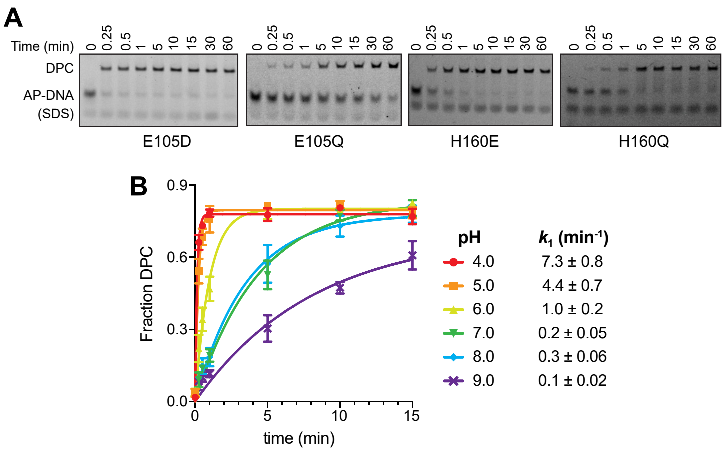

Figure S2. **A**. Representative denaturing PAGE gels of AP-DNA crosslinking by YedK point mutants. Bands were visualized by FAM fluorescence. **B**. Kinetics of crosslinking at different pH (mean ± SD, n=3). Reactions were performed at 18 °C. Rate constants derived from exponential fits to the curves are shown on the right and plotted in Fig. 1G.

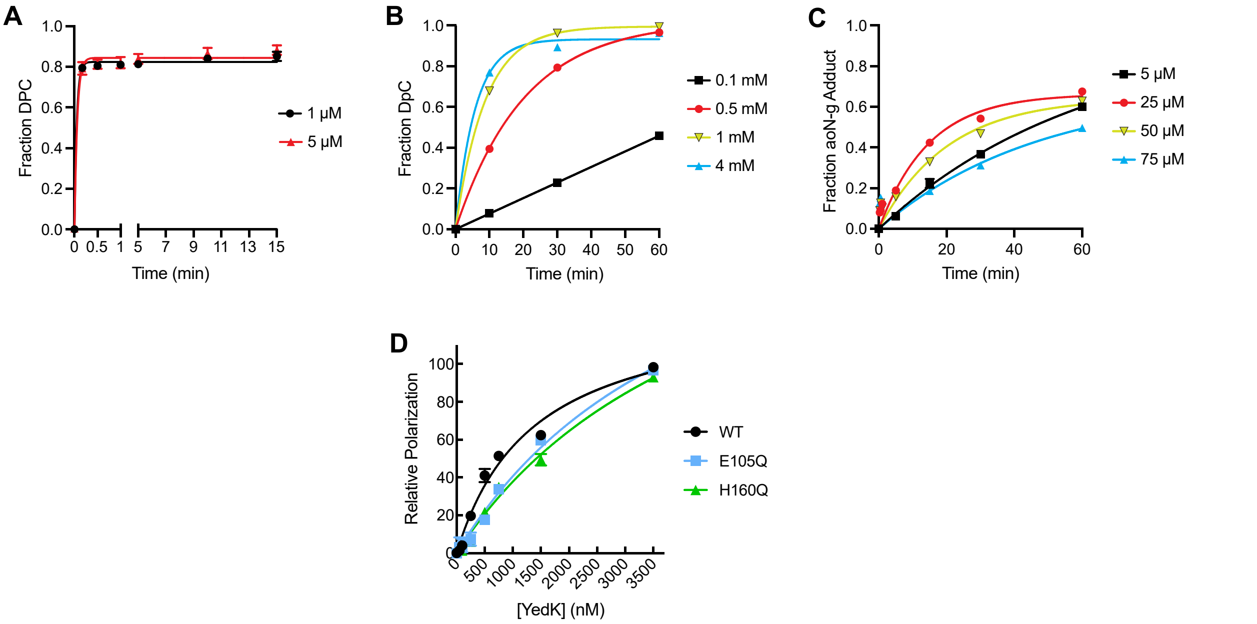

Figure S3. A-C. Determination of saturating conditions for crosslink formation using increasing concentrations of (A) YedK, (B) YedK peptide, and (C) aoN-g probe. **D**. Binding of formaldehyde-blocked YedK to ssDNA containing a 5′-FAM label and a centrally located tetrahydrofuran (THF) abasic site analog. Binding was monitored by a change in fluorescence polarization as protein was titrated against DNA. Dissociation constants (K_d) derived from non-linear least squares fit of the data are 1.4 µM ± 0.007 (WT), 4.5 ± 0.02 (E105Q), and 4.9 ± 0.02 (H160Q).

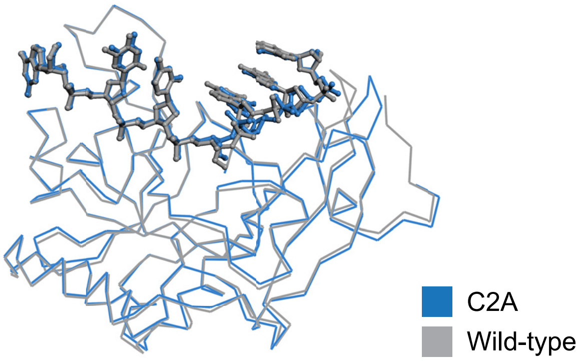

Figure S4. Superposition of YedK C2A trapped Schiff base (blue) and wild-type DPC (grey, PDB ID 6NUA) crystal structures.

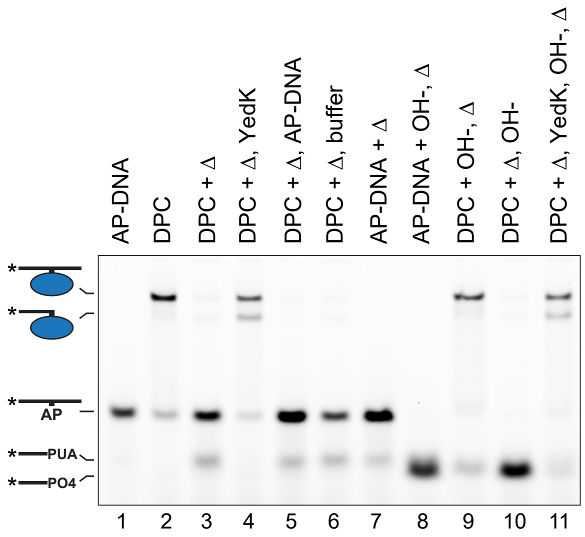

Figure S5. YedK DPC is refractory to strand breakage by alkaline pH. AP-DNA or DPC were reacted with hydroxide and heat (Δ) in the orders shown. Lanes 1-7 show that YedK DPC abolished after boiling can be re-formed with addition of fresh YedK but not DNA or buffer. Lanes 8-11 show that YedK DPC is refractory to cleavage by sodium hydroxide. DNA bands were visualized with FAM fluorescence. A cropped version of this gel containing lanes 1-7 is shown in Fig. 5A.

Supporting Tables

Table S1. YedK mutant crosslinking rates [a]

YedK mutant	k_1 (min^{-1})	k_2 (min^{-1})	Fold change (relative to WT)
WT [b]	≥ 10.3 ± 1.6		1
N75A	7.1 ± 1.5		0.7
E105A	4.1 ± 2.1	0.01 ± 0.01	0.4 | 0.001
E105D	6.3 ± 0.7		0.6
E105Q	1.2 ± 0.7	0.02 ± 0.02	0.1 | 0.002
H160A	1.1 ± 0.1		0.1
H160E	3.5 ± 1.1		0.3
H160Q	0.9 ± 0.1		0.1

[a] Reactions were carried out at pH 6.0, 25 ºC. Values are mean ± SD (n=3)
[b] Value for wild-type is a lower limit

Table S2. Sequences of oligodeoxynucleotides used

Name	Sequence (5'→3')	Figure(s)
FAM_U_Cy5	FAM-d(CGGGCGGCGGCAUAGGGCGCGGGCCTTTTTT)-Cy5	1B-G, 3E-F
FAM_U_20	FAM-d(TCTTCTGGTCUGGATGGTAGT)	5B-D
40_U	d(GGAATCTGACTCTTCTGGTCUGGATGGTAGTTAAGTCTTGT)	5B-D
FAM_U_35	FAM-d(ATGACTCTTCTGGTCUGGATGGTAGTTAAGTCTTGT)	2A-C, 2E, 4A, 4C, 5A, 5D
FAM_THF_15	FAM-d(TCTGGTC[THF]GGATGGT)	S2D
H36	d(GTCUGGA)	3B-C

Table S3. X-ray data collection and refinement statistics for YedK C2A DPC [a]

**Data collection**	
Space group	$P2_1$
Cell dimensions	
a, *b*, *c* (Å)	60.94 41.47 82.58
α, *β*, *γ* (°)	90.00, 95.65, 90.00
Resolution (Å)	50.00 - 1.83 (1.90 - 1.83)
R_{sym}	0.076 (0.667)
R_{meas}	0.101 (0.891)
$CC_{1/2}$	0.994 (0.507)
Avg. *I*/σ*I*	11.9 (1.4)
Completeness (%)	99.53 (96.06)
Redundancy	2.1 (2.1)
Wilson *B*-factor (Å^2)	19.8
**Refinement**	
Resolution (Å)	37.03 - 1.82 (1.87 - 1.82)
No. reflections	37,024 (3,583)
R_{work}	0.178 (0.254)
R_{free}[b]	0.226 (0.307)
No. atoms[c]	
Protein	3,500
DNA	246
Water	260
Other	0
Avg. *B*-factors[c,d] (Å^2)	
Protein	26.6
DNA	29.4
Water	27.8
Other	-
R.m.s. deviations	
Bond lengths (Å)	0.011
Bond angles (°)	1.109

[a] Statistics for the highest resolution shell are shown in parentheses.
[b] R_{free} was determined from the 5% of reflections excluded from refinement.
[c] Riding hydrogen atoms were not included in no. atoms or avg. *B*-factors.
[d] Equivalent isotropic *B*-factors were calculated in conjunction with TLS-derived anisotropic *B*-factors
